# The miR-26a-5p/IL-6 axis alleviates sepsis-induced acute kidney injury by inhibiting renal inflammation

**DOI:** 10.1080/0886022X.2022.2056486

**Published:** 2022-05-02

**Authors:** Yanhong Chen, Xu Zhou, Yanhong Wu

**Affiliations:** Department of Critical Care Medicine, Hunan Provincial People’s Hospital (The First Affiliated Hospital of Hunan Normal University), Changsha, China

**Keywords:** Acute kidney injury, miR-26a-5p, IL-6, inflammation, NF-κB

## Abstract

Sepsis-induced acute kidney injury (AKI) is a common and life-threatening complication in hospitalized and critically ill patients and has unacceptable morbidity and mortality rates. However, effective approaches for the diagnosis and treatment of septic AKI are still lacking. Here, we demonstrated significant increases in the miR-26a-5p levels in renal tubular cells of LPS-induced septic AKI models both *in vivo* and *in vitro*. Mechanistically, we provided evidence of the involvement of NF-κB in miR-26a-5p induction. LPS treatment of renal tubular cells led to the activation of NF-κB, and inhibition of NF-κB by TPCA-1 prevented the induction of miR-26a-5p. These results indicated that NF-κB was a key upstream factor for the induction of miR-26a-5p in septic AKI. Anti-miR-26a-5p enhanced the expression of IL-6 at both the protein and mRNA levels following LPS treatment. Furthermore, our luciferase microRNA target reporter assay verified that IL-6 is a direct target of miR-26a-5p. Blocking miR-26a-5p promoted renal inflammation and worsened kidney injury. Thus, our study indicated that the miR-26a-5p/IL-6 axis can alleviate sepsis-induced acute kidney injury by inhibiting renal inflammation. This mechanism may represent a therapeutic target for septic AKI.

## Introduction

Acute kidney injury (AKI) is a major kidney disease characterized by a rapid decline in renal function within a short period. Clinically, AKI is mainly caused by sepsis, ischemia–reperfusion (I/R), and various nephrotoxins [1]. Among these, sepsis is the main cause of AKI, accounting for nearly half of all cases of AKI [1,2]. Accordingly, septic AKI is a common and life-threatening complication in hospitalized and critically ill patients and has unacceptable morbidity and mortality rates [3,4]. Therefore, it is extremely urgent to elucidate the pathogenesis of septic AKI and find an effective treatment strategy. Recent studies have indicated that many factors are involved in the development of septic AKI, including microvascular dysfunction, inflammation, and metabolic reprogramming [4]. However, despite these findings advancing our understanding of the pathophysiology of septic AKI, effective approaches for its diagnosis and treatment are still lacking.

Inflammatory injury may play a key role in AKI [5–7]. Generally, circulating inflammatory cytokines directly affect renal tubular cells and are associated with an increased risk of mortality in AKI patients [8–11]. However, recent studies have demonstrated that renal resident tubular cells play dual roles in AKI [12]. Indeed, these cells not only express cytokine receptors but also release proinflammatory molecules [12]. Massive levels of inflammatory cytokines contribute to tubular cell apoptosis and ultimately contribute to AKI [12]. NF-κB, which consists of five subunits [RelA (p65), RelB, c-Rel, NF-κB1 (p50/p105), and NF-κB2 (p52/p100)], is reportedly the main proinflammatory transcription factor in septic AKI [13,14]. Generally, NF-κB is localized in the cytoplasm and binds to IκB-α, which prevents its translocation into the nucleus. Once stimulated, NF-κB translocates into the nucleus and triggers proinflammatory cytokine secretion [15].

MicroRNAs (miRNAs) are a group of small noncoding RNA molecules that are approximately 22 nucleotides long. They regulate gene expression mainly by blocking the translation of target mRNAs by targeting to their 3′-untranslated regions (UTRs) [1]. Decades of research have provided significant insights into the roles of miRNAs in the pathogenesis of human diseases, including kidney disease [16]. In AKI, ablation of Dicer (a key gene for microRNA biogenesis) in kidney proximal tubules induced renal ischemia/reperfusion injury resistance in mice, suggesting the critical role of miRNAs in AKI [17]. In septic AKI, Liu et al. demonstrated that NF-κB inhibited the expression of miR-376b in renal tubular cells, leading to the induction of NF-κBIZ, which provided a negative feedback mechanism to suppress NF-κB [11]. In another study, the authors also indicated that urinary miR-452 might be an effective biomarker for the early detection of AKI in septic patients [18]. Interestingly, both miR-376b and miR-452 were regulated by NF-κB in these two studies [11,18]. In fact, studies have verified that NF-κB not only regulates multiple miRNAs at the transcriptional level but is also a direct or indirect target of multiple miRNAs [19]. However, despite this progress, the mechanism by which miRNAs regulate septic AKI remains largely unclear. In particular, the specific miRNAs in septic AKI remain to be discovered.

The results herein indicated that miR-26a-5p was induced in an NF-κB-dependent manner in the renal tubular cells of mice with LPS-induced septic AKI. We found that miR-26a-5p could alleviate tubular cell death and kidney injury, exerting a protective effect against septic AKI. In addition, blocking miR-26a-5p aggravated kidney damage. Furthermore, we identified IL-6 as a direct target of miR-26a-5p. Taken together, the results of our study indicate that the miR-26a-5p/IL-6 signaling pathway alleviates kidney injury in septic AKI.

## Materials and methods

### Reagents

Antibodies were from the following sources: Anti-p65 (8242), anti-p-p65 (3033), anti-cleaved caspase-3 (9664), and anti-GAPDH (5174) were from Cell Signaling Technology; Anti-IL-6 (66146) was from Proteintech; Anti- F4/80 was from Servicebio (Wuhan China). The secondary antibody for immunoblot was from Thermo Fisher Scientific. Special regents were shown as follows: digoxigenin-labeled mmu–miR-26a-5p LNA probes and the Fluorescence *In Situ* Hybridization Kit were from Servicebio; miR-26a-5p mimic, anti-miR-26a-5p, and relative negative control oligonucleotide were from Ribo Biotechnology (Guangzhou, China); LPS was from Sigma, and TPCA-1 (A4602) was from APExBIO.

### Animals and septic AKI induction

Male C57BL/6 mice (7 weeks) were purchased from Slaccas Animal Laboratory (Changsha, China). All mice were acclimated to a 12-h light/dark cycle at 24 °C with 50% humidity and were given free access to food and water for at least 1 week before the experiments. The protocol was approved by the Institutional Animal Care and Use Committee. Septic AKI was induced by the intraperitoneal injection of LPS (10 mg/kg body weight). Control mice were injected with normal saline. The mice were euthanized after 24 h after LPS treatment and blood and kidney tissues were collected for further analysis. Serum IL-1β, IL-6 and TNF-α were measured by using ELISA Kits (CUSABIO, Wuhan, China) according to the manufacturer's instructions. In some experiments, the mice were administered anti-miR-26a-5p LNA (20 mg/kg) or NC oligonucleotide LNA *via* their tail vein.

### Renal morphological and functional studies

Kidney tissues were fixed with 4% paraformaldehyde and embedded in paraffin. Kidney tissue sections of 4 μm were then prepared. H&E staining was conducted to analyze renal histology. Serum creatinine and BUN were measured to evaluate renal function with reagents from BioAssay Systems (Hayward, CA).

### Fluorescence *in situ* hybridization (FISH)

Kidney tissues were collected and sliced into 4-μm-thick sections. The sections were permeabilized with 20 μg/ml proteinase K and then incubated with a prehybridization solution at 78 °C for 1 h. The prehybridization solution was removed, and the digoxigenin-labeled mmu-miR-26a-5p LNA probe was added overnight at 37 degrees. On the second day, the sections were washed and blocked with bovine serum albumin (BSA). Then, the sections were incubated with anti-digoxigenin-HRP at 37 °C for 1 h. CY3-TSA and DAPI were used to indicate the positive staining areas and cell nuclei, respectively.

### Cell culture

The Boston University mouse proximal tubular cell line (BUMPT) was used in this study. To establish the septic AKI cell model, the cells were treated with 100 μg/ml LPS for 24 h. Control cells were maintained in normal medium. In some experiments, TPCA-1 was added simultaneously with LPS at a final concentration of 50 μM.

### Western blotting

Equal amounts of protein samples were separated by 12% sodium dodecyl sulfate–polyacrylamide gel electrophoresis under reducing conditions and then transferred onto polyvinylidene difluoride membranes. After blocking with 5% skimmed milk at room temperature for 1 h, the membranes were incubated overnight (at 4 °C) with primary antibodies (The dilution for all the primary antibodies used in Western Blot was 1:1000). After washing, the membranes were incubated at room temperature for 1 h with HRP-conjugated anti-rabbit secondary antibodies (dilution: 1:5000). Protein bands were detected with an enhanced chemiluminescence kit (Thermo Fisher Scientific, 32106). For Western blotting, GAPDH was used as loading control to normalize the data.

### Quantitative real-time PCR

The quantitative real-time PCR was performed as described in other study [20]. Briefly, total RNAs were isolated from cultured BUMPT cells or kidney tissues with the TRIzol (Thermo Fisher Scientific). For qPCR analysis of miRNAs, 50 ng of total RNAs from each sample were reversely transcribed into cDNA by using the microRNA Reverse Transcription kit (Applied Biosystems), and qPCR was performed with a TaqMan miRNA assay kit (4440887; Applied Biosystems). For qPCR analysis of mRNAs, 1ug of total RNAs from each sample were reversely transcribed into cDNA by using an M-MLV Reverse Transcriptase cDNA Synthesis Kit (TaKaRa), and qPCR was performed with TB GreenTM Premix Ex Taq II reagent (TaKaRa). All PCR data were analyzed by LightCycler 96 SW 1.1 software, and relative levels were determined by the 2-ΔΔCt method. For miRNA analysis, miR-26a-5p was normalized to the level of U6 (internal control) to determine the ratios. The ratios of control mice were arbitrarily set as 1. For mRNA analysis, IL-1, IL-6 or TNF-α mRNA (Primers applied as shown in Table 1) was normalized to the level of GAPDH (internal control) to determine the ratios. The ratios of control mice were arbitrarily set as 1.

**Table 1.** Primers applied in qPCR.

	Sense primer (5′–3′)	Antisense primer (5′–3′)
IL-1β	AAAGCTTGGTGATGTCTGGT	TCTACACTCTCCAGCTGTAG
IL-6	CACCTCTTCAGAACGAATTG	GGATCAGGACTTTTGTACTC
TNF-α	CCACGCTCTTCTGCCTGCTG	CTGGAGCTGCCCCTCAGCTT
GAPDH	AGGTCGGTGTGAACGGATTTG	GGGGTCGTTGATGGCAACA

### Immunohistochemistry and cellular immunofluorescence analyses

For immunohistochemistry analysis, paraffin-embedded kidney sections were sequentially subjected to deparaffinization, hydration, and antigen retrieval. Then, the tissue sections were exposed to anti-IL-6 or F4/80 (1:200) at 4 °C overnight followed by exposure to a horseradish peroxidase (HRP)-conjugated secondary antibody for 1 h at room temperature. Signals of the antigen–antibody complexes were detected with a DAB Peroxidase Substrate Kit (Vector Laboratories) in accordance with the manufacturer’s instructions. For cellular immunofluorescence, fixed cells were permeabilized with 0.1% Triton X-100 and incubated in blocking buffer. The specimens were sequentially incubated with an anti-p65 (1:200) antibody overnight at 4 °C, Rhodamine-conjugated secondary antibodies for 1 h at room temperature, and DAPI (MilliporeSigma, D9542).

### TUNEL assay

TUNEL staining was performed to identify apoptotic cells in renal tissues using a reagent (12156792910) from Roche Life Science. Briefly, tissue sections were deparaffinized and pretreated with 0.1 M sodium citrate (pH 6.0) at 65 °C for 30 min and then incubated with a TUNEL reaction mixture for 1 h at 37 °C in a humidified, dark chamber. Positive staining with nuclear DNA fragmentation was detected by fluorescence microscopy.

### Luciferase microRNA target reporter assay

The target reporter assay was conducted as shown in other study [11,20]. Briefly, 3′-UTR of the mouse IL-6 gene was synthesized and inserted into the pMIR-REPORT Luciferase plasmid (Life Technologies).The plasmids were cotransfected with pMIR-REPORT β-gal control plasmid and 200 nM miR-26a-5p mimics into HEK293 cells. One day after the transfection, cell lysate was collected in reporter lysis buffer from the Luciferase assay system (promega). The luciferase activity was detected by microplate reader and then normalized with β-galactosidase activity.

### Statistical analysis

Data are expressed as the means ± SDs. Comparisons between two groups were evaluated by Student’s t test, and comparisons between multiple groups were evaluated by one-way ANOVA. *p* < 0.05 was considered significant. GraphPad Prism 7.0 was used for all calculations.

## Results

### The expression of miR-26a-5p is upregulated in renal tubular cells of mice with LPS-induced septic AKI

**Figure 1.**
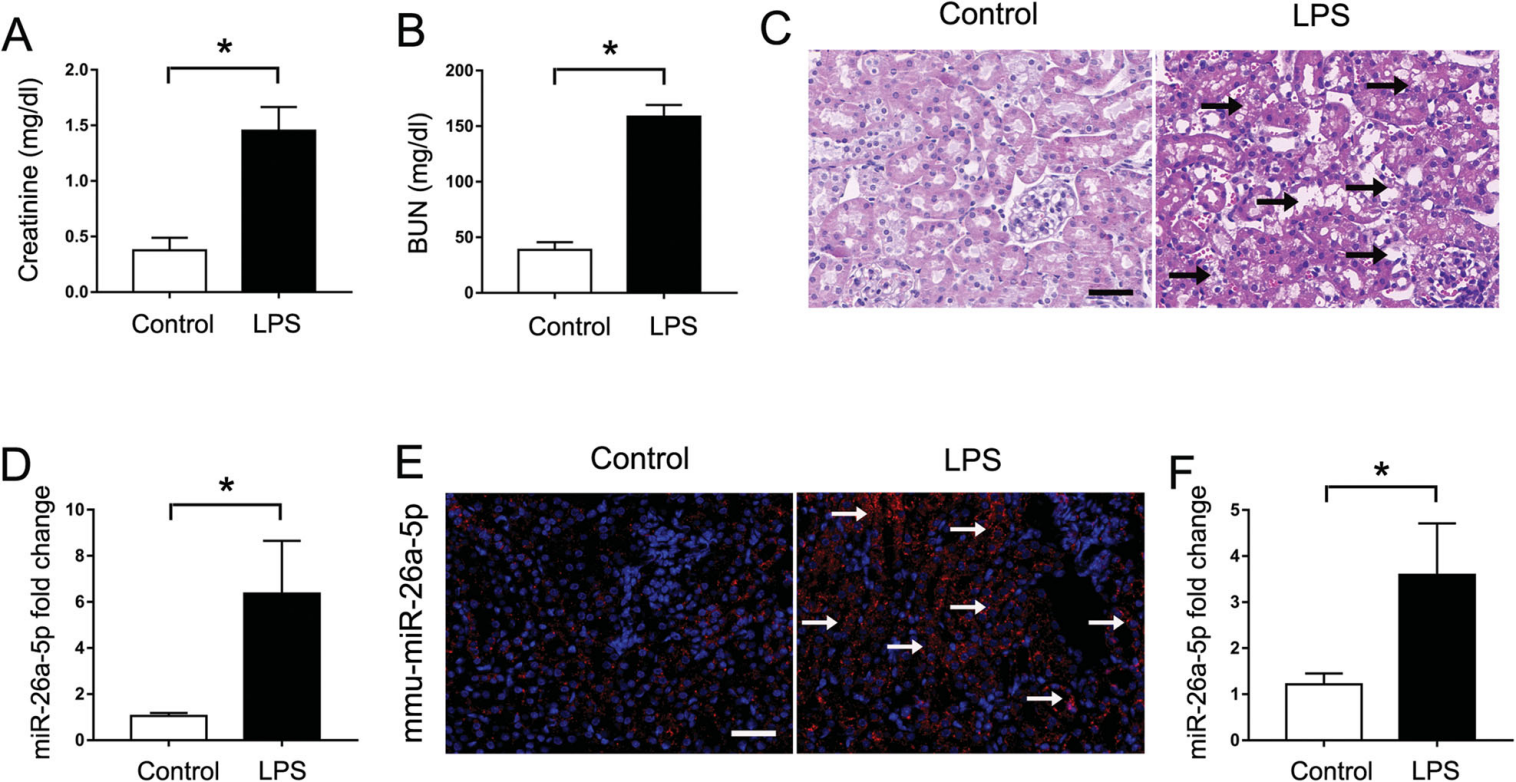
The expression of miR-26a-5p is upregulated in the renal tubular cells of mice with LPS-induced septic AKI. Eight-week-old male C57BL/6 mice were injected with 10 mg/kg LPS, and their kidney tissues were collected at 24 h after LPS treatment. Control mice were injected with normal saline. All the data are expressed as the mean ± SD (*n* = 6), *p < 0.05. (A) Serum creatinine. (B) Blood urea nitrogen (BUN). (C) Representative images of hematoxylin-eosin (HE) staining, Scale:50μm. (D) qPCR analysis of miR-26a-5p in the kidneys of mice treated with LPS or normal saline. U6 was used as the internal loading control. (E) *In situ* hybridization showing increased miR-26a-5p expression in renal tubules after LPS treatment, Scale:50μm. (F) qPCR analysis showed that miR-26a-5p was induced in LPS-treated BUMPT cells. BUMPT cells were treated with LPS (100 μg/ml). U6 was used as the internal loading control. All the data are expressed as the mean ± SD (*n* = 6), *p < 0.05.

In this study, we initially established a septic AKI mouse model by administering one dose of LPS (10 mg/kg) *via* injection. As shown in Figure 1(A,B), the levels of both serum creatinine and blood urea nitrogen (BUN) were increased at 24 h after LPS treatment compared with those in control mice. Consistently, histological analysis by HE staining also showed the most obvious kidney tissue injury (including nuclear abscission, tubule swelling, ablation of brush border, vacuolization of epithelial cells and so on) in the LPS-treated mice (Figure 1(C)). These data indicated the successful establishment of the septic AKI mouse model. Then, we collected renal tissues from LPS-treated and control mice to perform global miRNA profiling and found a series of miRNAs with altered expression (Tabel 2). Among these differentially expressed miRNAs, we finally confirmed the induction of miR-26a-5p by qPCR analysis (Figure 1(D)). Furthermore, fluorescence *in situ* hybridization (FISH) analysis revealed that the LPS-induced upregulation of miR-26a-5p mainly occurred in renal tubules (Figure 1(E)). In addition, we also provided evidence that miR-26a-5p was induced in cultured BUMPT cells (Figure 1(F)) and HK2 cells (Figure S1) subjected to LPS treatment. Taken together, these findings suggest that miR-26a-5p is induced in renal tubular cells in subjects with septic AKI.

**Table 2.** Microarray profiling of microRNA expression in LPS induced septic AKI.

Up-regulated miRNAs		Down-regulated miRNAs	
miRNAs	fold changes	miRNAs	fold changes
miR-34a-5p	22.8	miR-363	15.8
miR-26a-5p	22.5	miR-378b	15.7
miR-547-3p	21.7	miR-432	13.7
miR-8103	20.6	miR-453	12.8
miR-207	20.2	miR-466f	12.2
miR-211	19.8	miR-484	11.9
miR-21b	17.4	miR-670	11.4
miR-25-3p	16.5	miR-100-3p	8.6
miR-28b	16.4	miR-1187	7.8
miR-343	10.2	miR-133c	5.3

### NF-κB mediates the induction of miR-26a-5p in LPS-induced septic AKI

**Figure 2.**
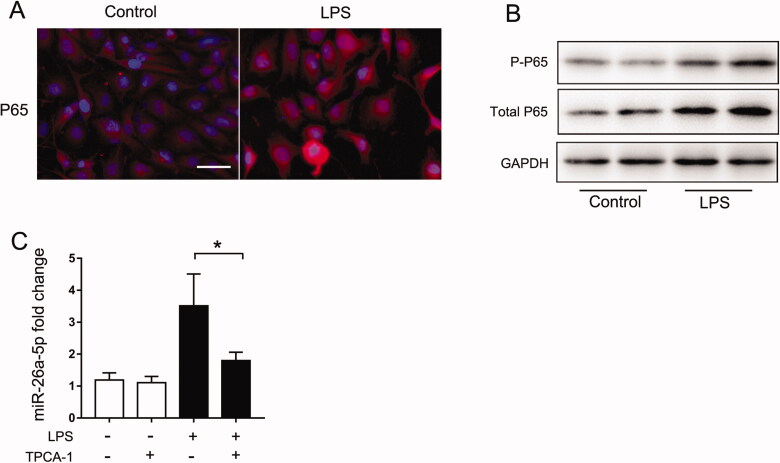
NF-κB mediates the induction of miR-26a-5p in LPS-induced septic AKI. BUMPT cells were exposed to LPS (100 μg/mL) for 24 h, and control cells were treated with PBS. (A) Immunofluorescence analysis of p65. The images were acquired with a fluorescence microscope, Scale:50μm. (B) Expression of total p65 and p-p65 was detected by Western blot, and GAPDH was used as the internal control. (C)qPCR analysis of miR-26a-5p showing the inhibitory effect of TPCA-1. BUMPT cells were treated with 50 μM TPCA-1 and then treated with LPS. U6 was used as the internal loading control. All the data are expressed as the mean ± SD (*n* = 4), *p < 0.05.

As described above, the NF-κB pathway plays an essential role in the expression of proinflammatory cytokines in resident renal cells [21,22]. In addition, NF-κB can also regulate multiple miRNAs transcriptionally [19]. To assess its involvement in miR-26a-5p induction, we evaluated NF-κB activation in LPS-treated BUMPT cells. Immunofluorescence staining (Figure 2(A)) showed that compared with the control group, stimulation with LPS increased NF-κB p65 expression and translocation into the nucleus. Immunoblot analysis further indicated that both the total p65 and p-p65 protein levels were significantly increased after LPS treatment (Figure 2B). These findings indicated the activation of NF-κB signaling in septic AKI. Then, we further examined whether the induction of miR-26a-5p depends on p65. TPCA-1, a commonly used inhibitor of p65, was used. Our results indicated that TPCA-1 completely blocked the induction of miR-26a-5p by LPS in BUMPT cells (Figure 2(C)). Collectively, these results suggest that NF-κB mediates miR-26a-5p induction in septic AKI. However, in another study conducted by Li et al. have shown reductions in TLR-2 & 4 and NF-κB P65 by miR-26a-5 mimic in the mouse alveolar macrophage severe pneumonia model, suggesting that miR-26a-5p regulates TLR/NF-κB signal pathway [23]. In our study, we did find miR-26a-5p had no effect on the NF-κB translocation in LPS treated BUMPT cells (Figure S2). We believe the difference between the two studies may be due to different cell has been used.

**Figure 3.**
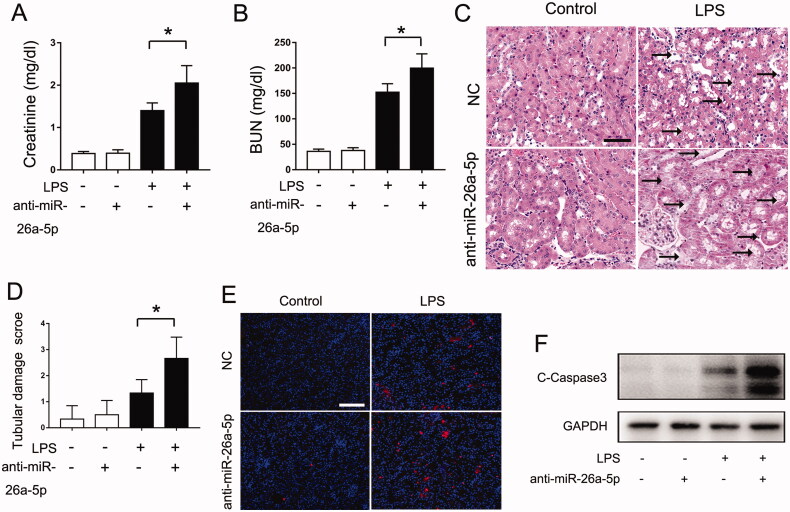
Blocking miR-26a-5p exaggerated LPS-induced septic AKI in mice. A nti-miR-26a-5p agent (20 mg/kg) or negative control (NC) was administered to male C57BL/6 mice through tail vein injection two days before LPS injection. The mice were then injected intraperitoneally with 10 mg/kg LPS, and their kidney tissues were collected at 24 h after the LPS injection. Control mice were injected with normal saline. All the data are expressed as the mean ± SD (*n* = 6), *p < 0.05. (A) Serum creatinine. (B) Blood urea nitrogen (BUN). (C) Representative images of hematoxylin-eosin (HE) staining, Scale Bar: 50 μm; (D) The graph shows semiquantitative tubular injury scores detected by HE staining; (E) Representative images of TUNEL staining, Scale Bar: 100 μm; (F) The expression of cleaved caspase-3 was detected by Western blot, and GAPDH was used as the internal control.

### Blocking miR-26a-5p exaggerated LPS-induced septic AKI in mice

To determine the role of miR-26a-5p in septic AKI, we assessed the effects of anti–miR-26a-5p on the LPS-induced septic AKI mouse model. Anti-miR-26a-5p did not cause kidney injury in control mice, but it significantly exaggerated tubular damage in LPS-induced septic AKI mice. As shown in Figure 3(A–D), anti-miR-26a-5p significantly increased the levels of serum creatinine and BUN and aggravated kidney tissue damage in LPS-treated mice. In addition, anti-miR-26a-5p promoted renal apoptosis as shown by the TUNEL assay and immunoblot analysis of cleaved-caspase 3 (Figure 3(E,F)). Taken together, these results indicate a protective role of miR-26a-5p in septic AKI.

### Blocking miR-26a-5p promotes renal inflammation in LPS-induced septic AKI mice

**Figure 4.**
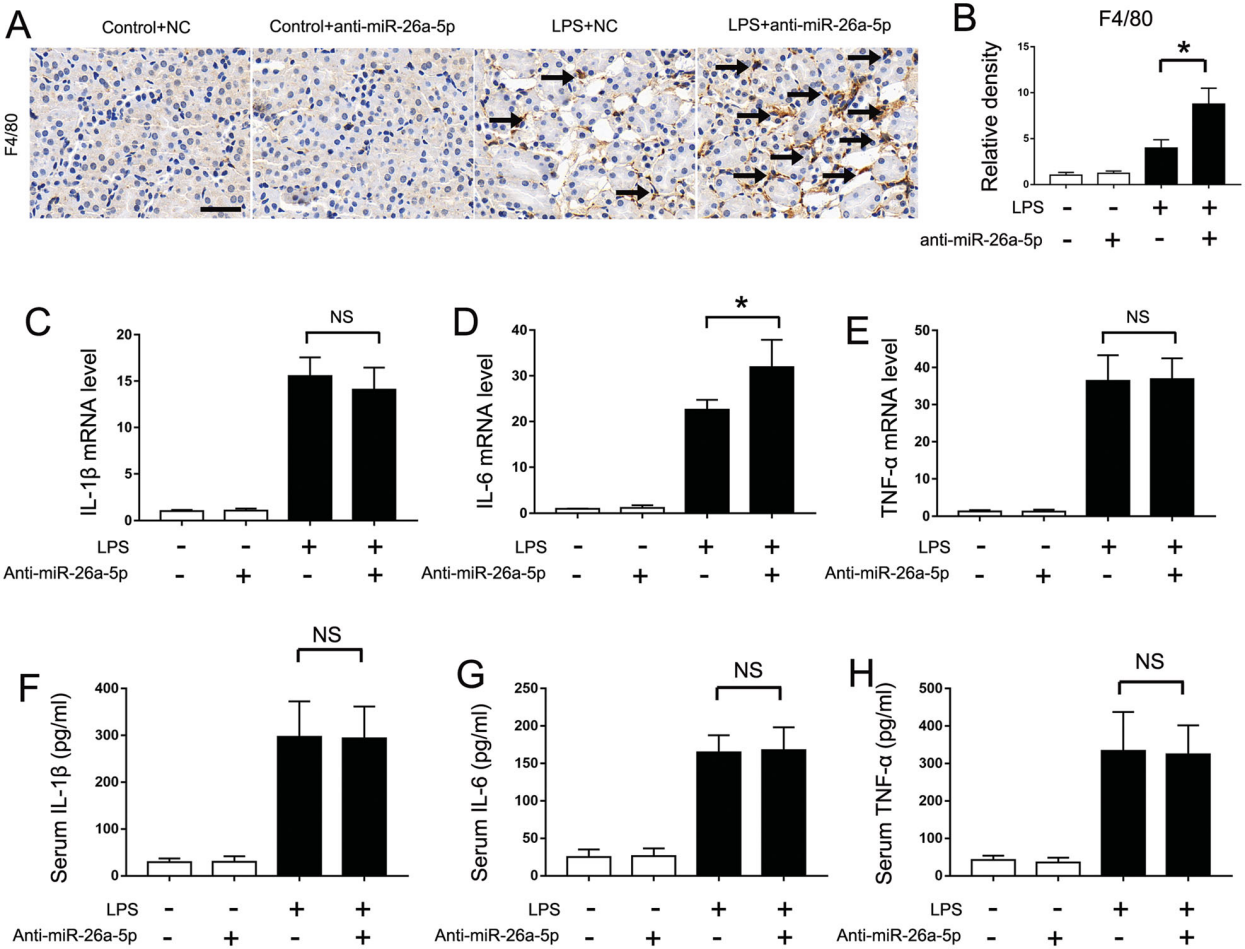
Blocking miR-26a-5p promotes renal inflammation in LPS-induced septic AKI mice. An nti-miR-26a-5p agent (20 mg/kg) or negative control (NC) was administered to male C57BL/6 mice through tail vein injection two days before the LPS injection. The mice were then injected intraperitoneally with 10 mg/kg LPS, and their kidney tissues were collected at 24 h after the LPS injection. Control mice were injected with normal saline. (A) Representative immunohistochemistry staining images of F4/80 to show macrophages, Scale bar:50μm；(B) The graph shows semiquantitative F4/80 expression detected by immunohistochemical; The data are expressed as the mean ± SD (*n* = 6), *p < 0.05; (C-E) qPCR analysis of IL-1β, IL-6 and TNF-α mRNA in septic AKI mice. The data are expressed as the mean ± SD (*n* = 4), *p < 0.05. NS: not statistically significant; (F-H) Serum IL-1β, IL-6 and TNF-α detected by ELISA. The data are expressed as the mean ± SD (*n* = 6), *p < 0.05. NS: not statistically significant.

As described above, inflammatory injury may play a key role in septic AKI. Thus, we further determined the effects of anti-miR-26a-5p on inflammation in the LPS-induced septic AKI mouse model. Immunostaining analysis of macrophages with the specific F4/80 antibody revealed more macrophage infiltration in the kidney tissues of LPS + anti-miR-26a-5p–treated mice than in those of mice treated with only LPS (Figure 4(A,B)). In addition, anti-miR-26a-5p enhanced the production of IL-6 in LPS-treated mice (Figure 4(D)). However, our results also indicated that anti-miR-26a-5p had no effect on IL-1β or TNF-α expression (Figure 4(C,E)). Of course, kidney injury induced by LPS is also a systemic inflammation. We then detected the circulating inflammatory factors through ELISA kit and found miR-26a-5p had no effect on circulating inflammatory factors during LPS treatment (Figure 4(F–H)). These findings show that miR-26a-5p alleviates septic AKI by inhibiting renal inflammation, especially by suppressing IL-6 expression.

### miR-26a-5p targets IL-6 in LPS-induced septic AKI

**Figure 5.**
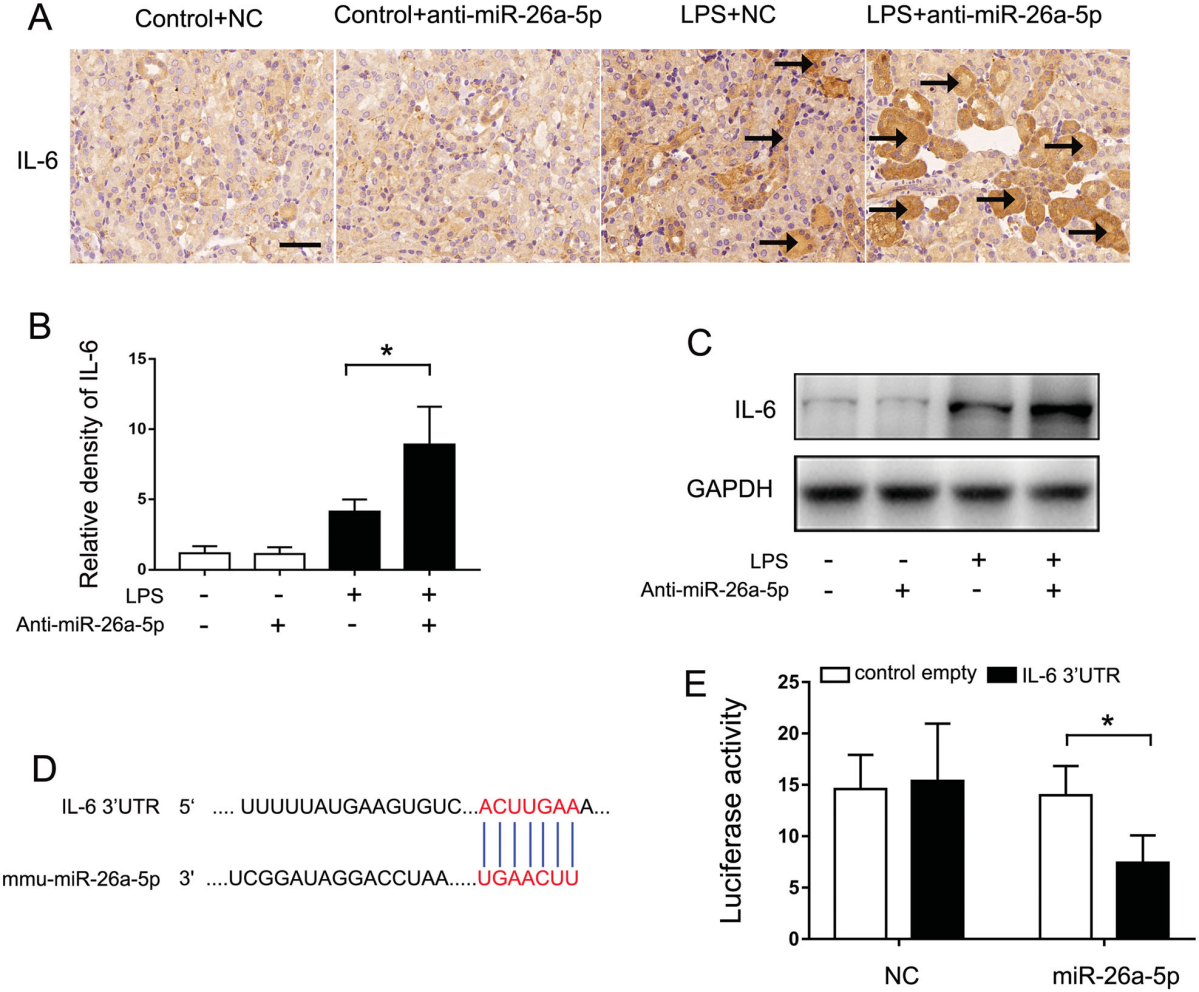
miR-26a-5p targets IL-6 in LPS-induced septic AKI. An nti-miR-26a-5p agent (20 mg/kg) or negative control (NC) was administered to male C57BL/6 mice through tail vein injection two days before LPS injection. The mice were then injected intraperitoneally with 10 mg/kg LPS, and their kidney tissues were collected at 24 h after the LPS injection. Control mice were injected with normal saline. (A) Immunohistochemistry showing the repressive effect of miR-26a-5p on IL-6 expression, Scale bar: 50 μm. (B) The graph shows semiquantitative IL-6 expression detected by immunohistochemical; The data are expressed as the mean ± SD (*n* = 6), *p < 0.05. (C) The expression of IL-6 was detected by Western blot, and GAPDH was used as the internal control. (D) The predicted miR-26a-5p binding site in 3’UTR of IL-6 mRNA. (E) MicroRNA target reporter assay of the IL-6 3’UTR. The putative miR-26a-5p binding sequence of the IL-6 3’UTR was cloned into the pMIR-REPORT plasmid to analyze luciferase activity after transfection of the miR-26a-5p mimic transfection; the activity was compared to that in the negative control group. β-gal was cotransfected for normalization. The data are expressed as the mean ± SD (*n* = 4), *p < 0.05.

The above results indicated that miR-26a-5p alleviates septic AKI by inhibiting renal inflammation, especially by suppressing IL-6 expression. To determine whether IL-6 is a target of miR-26a-5p, we first evaluated the effect of miR-26a-5p on the expression of IL-6 expression. Immunohistochemistry analysis found IL-6 was up-regulated in kidney tissues following LPS treatment, inhibiting miR-26a-5p would further promote the expression of IL-6 (Figure 5(A,B)). Our immunoblot analysis also confirmed this result (Figure 5(C)). Bioinformatic analysis identified a putative miR-26a-5p targeting sequence in the 3′-UTR of mouse IL-6 mRNA (Figure 5(D)). To determine if IL-6 is a direct target of miR-26a-5p, we performed luciferase microRNA targetreporter assay. Transfection of miR-26a-5p mimic suppressed luciferase expression from the reporter construct with IL-6 3′-UTR (Figure 5E). Collectively, these results indicate that IL-6 is a direct target gene of miR-26a-5p.

## Discussion

Sepsis accounts for nearly half of all AKI cases, has high morbidity and mortality rates, and lacks a treatment. In addition, the incidence of septic AKI continues to increase. Thus, septic AKI is a huge threat to human health [3,24]. However, the mechanism underlying septic AKI is still unclear. In this study, we found that miR-26a-5p expression in renal tubular cells was dramatically induced in an NF-κB-dependent manner in both *in vitro* and *in vivo* models of septic AKI. The induction of miR-26a-5p inhibited IL-6 expression, which finally alleviated renal tubular inflammation and protected against septic AKI. Blocking miR-26a-5p promoted renal inflammation and worsened kidney injury. Thus, our study indicates that the NF-κB/miR-26a-5p/IL-6 axis plays a protective role in septic AKI.

Recently, an increasing number of studies have indicated the crucial role of miRNAs in septic AKI. For example, Wang et al. indicated that miR-20a promoted kidney injury in septic rats through autophagy [25]. In addition, miR-106a has been indicated to aggravate septic AKI by targeting THBS2 [26]. However, despite this progress, the mechanism by which miRNAs regulate septic AKI remain largely unknown. In particular, the specific miRNAs in septic AKI remain to be discovered. In fact, miR-26a-5p has been confirmed to play a crucial regulatory role in diseases other than kidney diseases. For example, Shi et al. found that miR-26a-5p alleviated cardiac hypertrophy and dysfunction by targeting ADAM17 [27]. In addition, miR-26a-5p has been reported to play crucial roles in multiple neoplastic diseases [28,29]. However, in septic AKI, the role of miR-26a-5p is unclear. The results of the present study indicated that miR-26a-5p expression was significantly induced in kidney tubular cells in both *in vivo* and *in vitro* models of septic AKI. In addition, our *in situ* hybridization analysis revealed that miR-26a-5p was mainly localized in renal tubule cells of the renal cortex, and its induction also occurred in these cells following sepsis. Functionally, blocking miR-26a-5p led to increased cell death, to the induction of tubular injury and to the aggravation of septic AKI. These results indicate a protective role of miR-26a-5p induction in septic AKI.

Mechanistically, we provided evidence for the involvement of NF-κB in miR-26a-5p induction. NF-κB is a pleiotropic transcription factor that regulates the transcription of hundreds of genes involved in inflammation, immunity, apoptosis, cell proliferation, and differentiation [30]. In fact, studies have verified that NF-κB not only regulates multiple miRNAs transcriptionally but is also a direct or indirect target of multiple miRNAs [19]. In the present study, we demonstrated NF-κB activation in cultured BUMPT cells following LPS treatment by showing its phosphorylation and accumulation in the cell nucleus. Importantly, miR-26a-5p induction by LPS was completely suppressed by TPCA-1, a pharmacological inhibitor of the NF-κB signaling pathway. Taken together, these results showed that NF-κB was a key upstream factor for the induction of miR-26a-5p in septic AKI.

To elucidate the mechanism by which miR-26a-5p contributes to septic AKI, we investigated potential targets, identifying IL-6 as a direct target. IL-6 is a proinflammatory cytokine, and its production is triggered within minutes of numerous systemic insults, such as surgery, trauma, and sepsis, as part of the systemic inflammatory response syndrome (SIRS), which also occurs during AKI [31]. An increasing number of studies have indicated that IL-6 is a crucial molecule that promotes kidney injury and that inhibition of IL-6 can alleviate AKI [32]. For example, Zhao et al. indicated that inhibition of IL-6/sIL-6R axis activation attenuated sepsis-associated AKI [33]. In addition, suppression of IL-6/STAT3 signaling suppressed acute kidney injury-induced interstitial fibrosis and glomerulosclerosis [34]. In this study, anti-miR-26a-5p enhanced the expression of IL-6 at both the protein and mRNA levels following LPS treatment. Furthermore, our luciferase microRNA target reporter assay verified IL-6 as a direct target of miR-26a-5p. As described above, rather than being a mere victim of inflammation, renal tubular cells propagate intrarenal inflammation in septic AKI [12,35]. Thus, inhibition of IL-6 mainly inhibits renal inflammation and ultimately attenuates septic AKI. In fact, miR-26a-5p is a molecule that is highly involved in inflammatory response. A study conducted by Li et al. indicated that miR‑26a‑5p could alleviates LPS induced acute lung injury by targeting the connective tissue growth factor [36]. Besides, miR-26a-5p also could attenuate the inflammation of diabetic kidney disease through targeting CHAC1 [37]. Further, Cat et al. demonstrated that inhibition of Long Non-Coding RNA Small Nucleolar RNA Host Gene 5 (SNHG5) might mitigate HG-induced renal tubular damage *via* targeting miR-26a-5p [38].

Generally, NF-κB is a key to the proinflammatory response in a variety of cell types, including renal resident cells [15,22,39]. Normally, IκBs bind with NF-κB to mask their nuclear localization signals and keep them sequestered in the cytoplasm. In response to various cytokines, inflammatory molecules, and stress signals, IKKs are activated by phosphorylate IκB, resulting in the release and translocation of NF-κB into the nucleus for the transcription of various inflammatory genes [30]. Thus, even though the NF-κB induced miR-26a-5p induction in septic AKI could attenuate renal inflammation, it does not mean NF-κB has anti-inflammatory effects. In fact, the NF-κB induced miR-26a-5p induction in septic AKI must as a negative regulator of NF-κB. In other study, Liu et al. also demonstrated that NF-κB mediated miR-376b down-regulation could inhibit renal inflammation and cell apoptosis, which ultimately attenuate septic AKI [11]. In that study, NF-κB mediated miR-376b down-regulation in septic AKI also served as a negative regulator of NF-κB [11]. Thus, I believe that NF-κB serve upstream of an anti-inflammatory miRNA may be an adaptive self-protection phenomenon in response to kidney injury.

In conclusion, the present study demonstrated the NF-κB-mediated induction of miR-26a-5p in renal tubular cells in septic AKI. In addition, miR-26a-5p induction was shown to lead to the inhibition of IL-6, which ultimately attenuated renal inflammation. Thus, our study indicated that the miR-26a-5p/IL-6 axis alleviates sepsis-induced acute kidney injury by inhibiting renal inflammation. This mechanism may represent a therapeutic target for septic AKI.

## Supplementary Material

Supplemental Material

## Data Availability

The data that support the findings of this study are available from the corresponding author, [YW], upon reasonable request.
