# Supplementary material for: The miR-26a-5p/IL-6 axis alleviates sepsis-induced acute kidney injury by inhibiting renal inflammation
Source: Ren Fail. 2022 May 2;44(1):551–61. doi: 10.1080/0886022X.2022.2056486 (PMC9067948; doi:10.1080/0886022X.2022.2056486)
Supplement: Supplemental Material [file IRNF_A_2056486_SM9603.pdf]

The miR-26a-5p/IL-6 axis alleviates sepsis-induced acute kidney injury by inhibiting renal inflammation

Yanhong Chen[1], Xu Zhou[1], Yanhong Wu[1, *]

[1] Department of Critical Care Medicine, Hunan Provincial People's Hospital (The First Affiliated Hospital of Hunan Normal University), Changsha, 410005, Hunan Province, China.

*** Correspondence Author:** Yanhong Wu

Department of Critical Care Medicine, Hunan Provincial People's Hospital (The First Affiliated Hospital of Hunan Normal University), Changsha, 410005, Hunan Province, China (dellawyh @126.com).

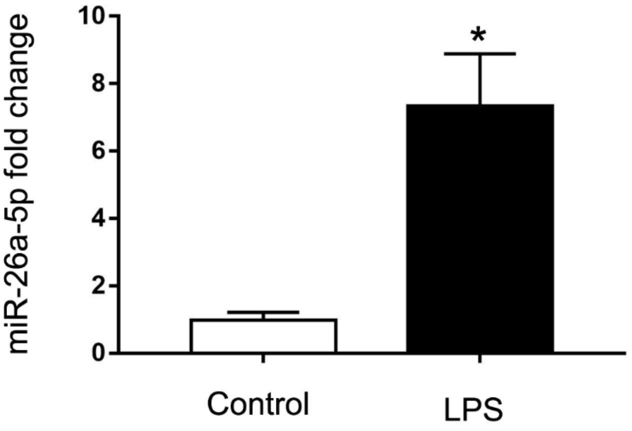

Figure S1: qPCR analysis showed that miR-26a-5p was induced in LPS-treated HK2 cells (Human renal tubular cells). HK2 cells were treated with LPS (100 µg/ml) for 24 hours. Control cells were maintained in normal medium. miR-26a-5p was normalized to the level of U6 (internal control) to determine the ratios. The ratios of control mice were arbitrarily set as 1. All the data are expressed as the mean ± SD (n=6), *P<0.05.

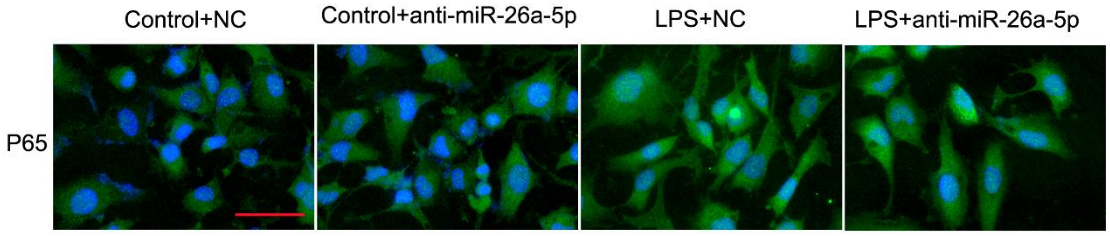

Figure S2: miR-26a-5p had no effect on NF-κB activation in BUMPT cells during LPS treatment. BUMPT cells were transfected with 200 nM anti-miR-26a-5p LNA or negative control sequences (NC), and then treated with 100µg/mL LPS for 24 hours. Immunofluorescence showing nuclear translocation of p65/NFKB in BUMPT cells after LPS treatment. Images were collected by laser scanned confocal microscopy. Scale bar, 50 µm.
